# Microscopic Origin of Charge Transfer at the Organic Semiconductor/MoO_3_ Hybrid Interface

**DOI:** 10.1021/acs.jpcc.5c05641

**Published:** 2025-10-07

**Authors:** Max Niederreiter, Maximilian Lasshofer, Francesco Presel, Giovanni Zamborlini, Luca Floreano, Luca Schio, Nadia C. Mösch-Zanetti, Svetlozar Surnev, Peter Puschnig, Martin Sterrer

**Affiliations:** † NAWI Graz, Institute of Physics, 27267 University of Graz, Universitätsplatz 5, 8010 Graz, Austria; ‡ CNR-Istituto Officina dei Materiali (IOM), Laboratorio TASC, Basovizza SS-14, Km 163.5, Trieste 34012, Italy; § University of Graz, NAWI Graz, Institute of Chemistry, Universitätsplatz 5, 8010 Graz, Austria

## Abstract

Molybdenum trioxide (MoO_3_) is widely utilized as an interfacial layer in organic electronic devices due to its high work function and favorable energy level alignment with organic semiconductors. While its role in facilitating hole injection has been extensively studied, the microscopic mechanisms underlying charge transfer at MoO_3_/organic interfaces remain elusive. Here, we investigate the interaction between 2H-phthalocyanine (2H-Pc) and ultrathin MoO_3_ films grown on Pd(100) as a model system to explore the microscopic origin of charge transfer from the organic layer to the oxide substrate. Using a combination of scanning tunneling microscopy/spectroscopy, X-ray photoemission spectroscopy, near-edge X-ray absorption fine structure, work function measurements, and density functional theory, we find clear evidence for integer charge transfer from the molecules into the substrate, resulting in positively charged molecules in both upright and flat adsorption geometries. The electronic signatures of charging are accompanied by distinct SOMO–SUMO gaps, with upright molecules exhibiting a small gap (∼0.4 eV), while flat-lying molecules show a significantly larger gap (∼1.5 eV) owing to reduced electronic screening. These findings provide atomically resolved insight into charge transfer and highlight how adsorption geometry and local dielectric environment govern the electronic structure of hybrid interfaces.

## Graphical abstract

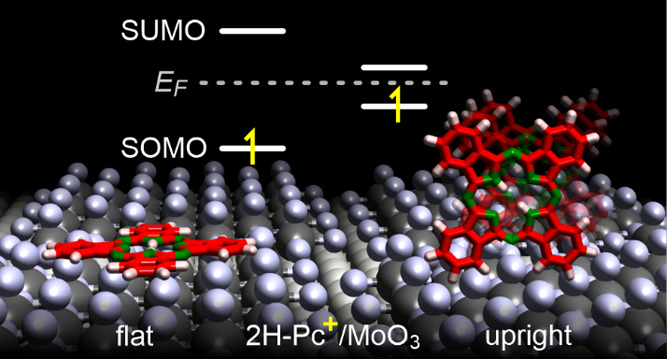

## 1 Introduction

Molybdenum trioxide (MoO_3_) is widely employed in organic electronic devices due to its ability to modify interface energetics. It plays a crucial role in organic light-emitting diodes, photovoltaics, and field-effect transistors.^1–5^ Owing to its large bandgap and optical transparency, MoO_3_ has also been considered for integration with silicon-based devices.^6^ While MoO_3_ can act as a hole transport layer (HTL) or a p-type dopant, its most important application is as an interfacial layer between metal electrode and organic hole transport layer, where it serves to reduce the barrier for hole injection. Since the discovery that MoO_3_, among other transition metal oxides, enhances the performance of optoelectronic devices,^7^ its specific role has been the subject of intense investigation.

One of the key properties that make MoO_3_ particularly suitable for such applications is its high work function of approximately 6.7 eV.^8^ This value aligns favorably with the ionization potential of typical organic HTL materials, enabling an energetically efficient alignment between the highest occupied molecular orbital (HOMO) of the organic material and the Fermi level (E_F_) of the substrate.^9–12^ Additionally, MoO_3_ exhibits a low-lying conduction band minimum, which gives it n-type semiconductor characteristics. This is believed to facilitate electron extraction from the HOMO of the organic material via the MoO_3_ conduction band, thereby enhancing hole injection.^13,14^ Experimental studies of MoO_3_/organic interfaces have revealed a pronounced decrease in the work function of MoO_3_ upon deposition of organic molecular layers.^10–13,15–19^ This is accompanied by the formation of a large interface dipole and HOMO-level pinning at the Fermi level, which are distinct signatures indicating charge transfer from the organic HOMO to the substrate, leading potentially to the formation of molecular cations.^20^ Additionally, investigations of various oxide/organic combinations have shown that such charge transfer phenomena follow a universal trend primarily governed by the work function of the oxide and the ionization potential of the organic material.^11^ While this has led to a generally consistent understanding of the role of high work function oxides at interfaces with organic semiconductors, the direct experimental confirmation of the charge transfer mechanism is challenging.

Charge transfer at molecule–substrate interfaces is generally classified as either fractional or integer, depending on the interaction strength.^21,22^ Strong interaction, such as on metal surfaces, typically leads to fractional charge transfer via hybridization of molecular and substrate states. In contrast, weakly interacting interfaces, such as organic/organic or oxide/organic systems, are often described by the integer charge transfer (ICT) model, which involves electron tunneling without significant orbital hybridization.^22^ In both cases, charge transfer results in the pinning of molecular energy levels to the substrate Fermi level. It occurs if charge is either transferred from the substrate to the molecule, corresponding to the situation when the molecules′ lowest unoccupied molecular orbital (LUMO) gets filled and pinned to *E*_F_, or, vice versa, when charge is transferred from the molecular HOMO to the substrate, in which case the HOMO gets pinned to *E*_F_.^23–26^ In the case of ICT, the pinning is accompanied by the splitting of the respective orbital into a singly occupied and singly unoccupied molecular orbital (SOMO and SUMO), derived from the original HOMO or LUMO.

The unambiguous identification of SOMO/SUMO states thus serves as the definitive signature of ICT.^27,28^ However, verifying this splitting is experimentally and theoretically challenging. The most employed techniques such as ultraviolet photoemission spectroscopy (UPS) and work function measurements provide indirect evidence of ICT through level pinning and interface dipoles but cannot resolve the orbital character of the frontier electronic states. Photoemission orbital tomography (POT) offers access to the symmetry of occupied frontier orbitals,^29^ yet fails to probe unoccupied states, and quantification of orbital occupation remains nontrivial. Scanning tunneling microscopy and spectroscopy (STM/STS), in contrast, can directly probe the local density of states (LDOS) in both occupied and unoccupied regions near the Fermi level,^30^ making it particularly suited for the identification of SOMO/SUMO pairs.^31^ However, both POT and STM/STS require atomically well-defined surfaces, which are often not accessible in real device structures. For this reason, one typically uses model systems such as metal or semiconductor single crystals, or, if wide-gap semiconductors or insulators are the subject of investigation, ultrathin, single-crystalline films of oxides or other dielectrics on metal surfaces.

Our recent combined STM/POT work on model systems consisting of ultrathin MgO(001) films on Ag(001) has demonstrated that charge transfer into monolayers of organic semiconductors can be characterized and controlled on the atomic level.^27,32–35^ These studies revealed how the formation of SOMO/SUMO states and the degree of charging can be systematically modulated by work function tuning. Due to the specific electronic properties of the used substrates and organic molecules, most of the work reported on ICT on model systems focused on charge transfer into the organic molecules, that is, occupation of the molecular LUMO. In contrast, the reverse ICT process, from molecules to the oxide substrate, thereby emptying the HOMO, is less-well explored, most likely because model systems with sufficiently high work function, which are required to induce the charge transfer, were unavailable.

In this work, we address the case of integer charge transfer from an organic overlayer into the substrate by investigating the interaction of 2H-phthalocyanine (2H-Pc), a model organic semiconductor, with ultrathin MoO_3_ layers grown on Pd(100) representing the technologically important MoO_3_ interlayers in optoelectronic devices. We probe the geometrical, electronic and charge transfer properties using a combination of work function measurements, near-edge X-ray absorption fine structure spectroscopy (NEXAFS), X-ray photoemission spectroscopy (XPS), scanning tunneling microscopy and spectroscopy (STM/STS), and density functional theory (DFT). In addition to providing direct proof for single integer charge transfer from the HOMO of 2H-Pc into the MoO_3_ substrate, we show how molecules in different geometries can be stabilized and how this affects their electronic properties.

## 2 Methods

### 2.1 Experimental Details

The Pd(100) single crystal was cleaned by several cycles of Ar^+^ ion bombardment and annealing to 700 °C. Molybdenum oxide MoO_3_ monolayers were prepared by depositing (MoO_3_)_3_ clusters from an e-beam evaporator at room temperature, and subsequent oxidation at 350 °C and in 1 × 10^–6^ mbar of O_2_ for 10 min, as described in more detail elsewhere.^36–38^ The oxide film morphology is very sensitive to the post oxidation parameters, it can, however, be verified easily using LEED, where the c(2 × 2) structure is clearly visible for well-ordered films, which we did to ensure a proper preparation. 2H-Pc was deposited from a Knudsen cell evaporator with the deposition rate being monitored by a quartz microbalance.

XPS and NEXAFS measurements were performed at the ALOISA beamline at Elettra Sincrotrone Trieste, where XPS was used to verify proper oxide stoichiometry, oxide and molecular film thicknesses and for work function measurements (Supporting Information SI.0). NEXAFS N and C K-edge measurements were used to determine the molecular orientation w.r.t the substrate surface. In the ALOISA setup, XPS was measured with the sample at a grazing incidence of 4° and a near-normal emission. The hemispherical analyzer (acceptance angle fwhm ∼ 2°) is equipped with a 2D delay-line detector and provides a kinetic energy resolution of 1% of the pass energy. The NEXAFS linear dichroism was measured by rotating the sample around the photon beam axis at constant grazing angle of 6°, thus switching the scattering plane from transverse to the electric field (TE, corresponding to strictly s-polarization) to transverse to the magnetic field (TM, corresponding to near p-polarization).^39^ NEXAFS spectra were measured in partial electron yield by means of a channeltron equipped with a front grid polarized to −250 V (C K-edge) and −370 V (N K-edge) to repel low energy secondary electrons. The photon energy resolution was set to 80 and 100 meV for C and N, respectively; the absolute energy calibration (±10 meV) of the spectra was performed by reference to the gas absorption spectra of CO and N_2_, as described in ref 40. The NEXAFS spectra were normalized to the spectra measured on the MoO_3_ overlayer before molecular deposition, as well as to the photon flux, following the protocol described in ref 41.

STM and STS measurements were done in a separate UHV system. The low-temperature STM using electrochemically etched tungsten tips was operated at 77 K for all measurements presented in this study. The bias voltage (*V*_b_) was applied to the sample. d*I*/d*V* spectra were recorded using a lock-in amplifier (*f*_mod_ = 2.345 kHz, *A*_mod_ = 25 mV).

### 2.2 Computational Details

We have modeled the interface of 2H-Pc with MoO_3_/Pd­(100) by performing density functional theory (DFT) calculations by using a repeated slab approach and employing the VASP code.^42,43^ For the MoO_3_/Pd­(100) substrate, we use the same setup as described in a previous publication,^37^ that is, a five-layer thick Pd slab in a (sqrt(2) × sqrt(2))-Pd(100) cell and the experimental Pd lattice parameter of *a* = 3.89 Å with the MoO_3_ units atop. For the 2H-Pc/MoO_3_/Pd­(100) interface with flat lying molecules, we construct a (4 × 4) supercell of the MoO_3_/Pd­(100) substrate containing one 2H-Pc molecule, while for the interface with upright-standing molecules, we construct a supercell with the epitaxial matrix ((6, −3), (1, 2)), which can accommodate four upright-standing 2H-Pc molecules in a herringbone arrangement as seen in STM. Exchange–correlation effects are treated via the Perdew–Burke–Ernzerhof generalized gradient approximation (PBE-GGA)^44^ with a Grimme D3 van der Waals correction.^45^ For the Pd-d electrons, we have added an effective Hubbard-U parameter of 4 eV using the Dudarev ansatz.^37,46^ In the geometry optimization, all atoms except for the bottom three Pd layers are allowed to relax until all atomic forces were below 0.01 eV/Å. A vacuum layer of at least 15 Å is used and a dipole layer has been added to account for different work functions at the bottom and top sides of the slab.^47^ We employ the projector augmented wave method with a plane wave cutoff of 500 eV, and sample the Brillouin zone with Γ-centered grids of 4 × 4 × 1 and 3 × 6 × 1 for the cell containing the flat lying and upright standing molecules, respectively.

To account for the effect of intermolecular screening on the ionization potential (IP) and electron affinity (EA) of single-positively charged 2H-Pc molecules, we have considered clusters containing up to eight molecules arranged in the herringbone pattern of upright standing molecules. For each cluster (see Supporting Information SI.5), we have computed the total energies of the cation, E­(N-1), the neutral system E­(N) and the di-cation, E­(N-2), and use the following relation to compute the IP, the EA and the fundamental gap E_gap_ of the cationic 2H-Pc:

IP=E(N−2)−E(N−1)

EA=E(N−1)−E(N)

$$E_{\mathrm{gap}}=\mathrm{IP}-\mathrm{EA}$$

For these DFT calculations, we have also employed the VASP code using a large cubic box of 38 × 38 × 38 Å^3^ and Γ-point sampling. Note that for these calculations, no further geometry relaxations have been performed, thus the reported values correspond to vertical IPs and EAs, respectively.

The computational data generated during this study are available in the NOMAD repository.^48,49^

## 3 Results and Discussion

The focus of the present study is on integer charge transfer from an organic semiconductor molecule into a suitable substrate with high work function. A rather straightforward and experimentally simple method to confirm charge transfer at such organic/inorganic interfaces is the study of work function changes (ΔΦ) resulting from the formation of charge transfer dipoles upon adsorption.^22^ While on metal surfaces the concomitant push-back effect often prevents meaningful conclusions about charge transfer from ΔΦ changes, this effect is largely absent if, as in the present case, an insulating decoupling layer is introduced.^27^ Here, ΔΦ changes result predominantly from charge transfer dipoles, and, if present, from orientation-dependent molecular dipoles.

Starting with a clean MoO_3_/Pd­(100) sample, which exhibits a high work function of 6.7 eV,^37,38^ the work function initially strongly decreases up to a 2H-Pc coverage of 0.2 ML and then continues to decrease with smaller slope up to monolayer coverage, where the work function change ΔΦ amounts to ∼−2 eV (Figure 1, and Supporting Information SI.1). Note that we define monolayer coverage as the full coverage of the MoO_3_ surface with upright standing 2H-Pc molecules (see below and Supporting Information SI.2). A further decrease, again with smaller slope, is noted at coverages exceeding 1 ML. In the light of the integer charge transfer model, a decrease of the work function is generally an indication of a charge transfer from the molecule into the substrate with electrons extracted from the molecular HOMO, which is then pinned to the substrates Fermi level, *E*_F_.^22^ Thus, our findings for the present system imply that the energy level alignment favors an emptying of the 2H-Pc′s HOMO. According to the capacitor model, which is often used to describe integer charge transfer at weakly interacting interfaces, the work function is expected to approach a constant value when the interface potential is equilibrated via charge transfer.^50^ While such behavior has indeed been observed in previous studies,^33^ for the present system the progression of ΔΦ with increasing coverage (Figure 1) deviates from the expected behavior. This indicates that, in addition to mere charge transfer, other effects that influence the interfacial dipole, such as molecular reorientation or intrinsic molecular dipoles, are also active. Additionally, for thicker organic semiconductor layers, band bending as a result of potential energy drop across the layers has been invoked and might also play a role in the present system.^25^ However, here we will focus on the charge transfer in the first interfacial layer and to learn more about the evolution of the 2H-Pc/MoO_3_ interface, we have studied the growth and orientation of 2H-Pc on the MoO_3_/Pd­(100) thin film substrate with STM and NEXAFS.

**Figure 1:**
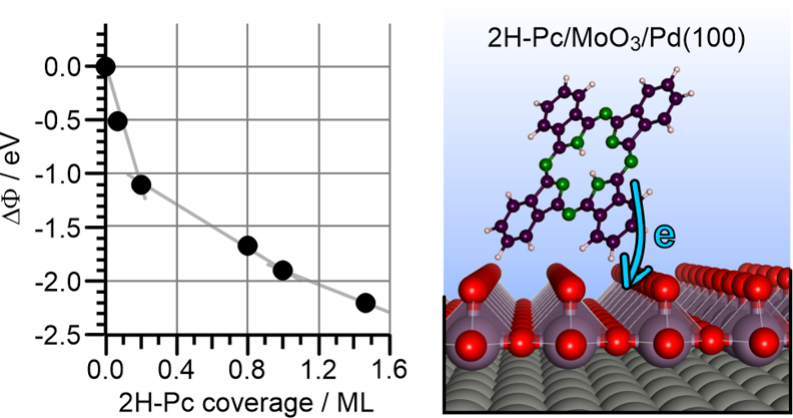
Work function change (ΔΦ) of MoO_3_/Pd­(100) with increasing amount of deposited 2H-Pc. ΔΦ = 0 refers to the work function of the clean substrate (Φ­(MoO_3_/Pd­(100)) = 6.7 eV). The strong decrease in work function indicates charge transfer from 2H-Pc into the substrate, as illustrated in the schematic.

An STM image of the pristine MoO_3_ film is shown in Figure 2a. As reported previously, the monolayer film consists of square islands separated by antiphase domain boundaries.^36,37^

**Figure 2:**
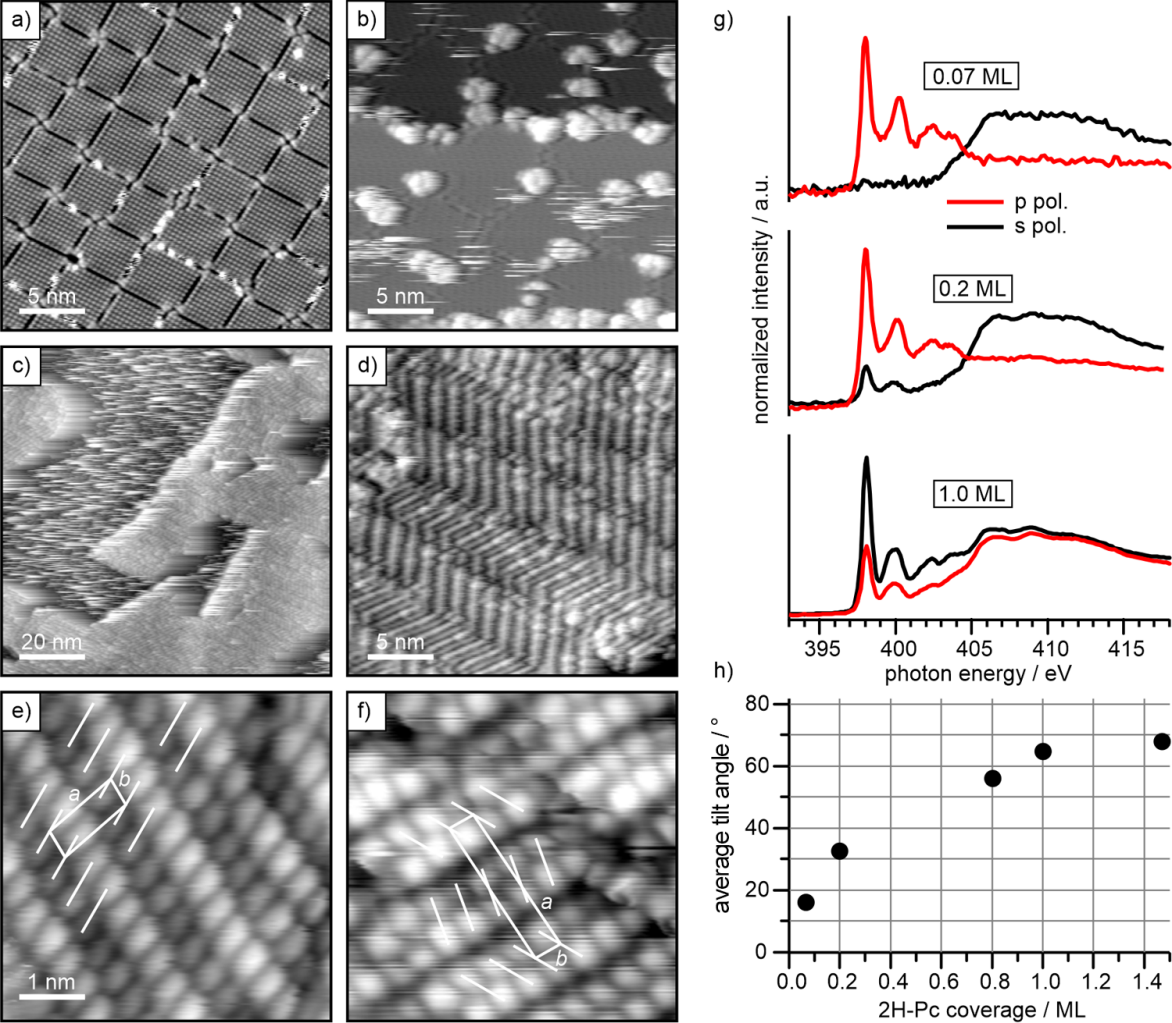
2H-Pc monolayer growth on MoO_3_/Pd­(100). STM images of (a) the clean MoO_3_ film and after deposition of (b) 0.07 ML, where flat 2H-Pc molecules predominantly adsorb at defect sites, and (c) 0.5 ML 2H-Pc, where islands of standing 2H-Pc molecules have formed. The STM image in (d) was obtained on top of an island and shows the stripe-like arrangement of 2H-Pc molecules. Better resolved images in (e, f) reveal details about molecular stacking and unit cells (see text for details). (g) N K-edge NEXAFS spectra for increasing coverage of 2H-Pc on MoO_3_/Pd­(100). (h) Average tilt angle of 2H-Pc molecules as a function of coverage. Tunneling conditions: (a) +1.3 V, 110 pA; (b) −1.4 V, 7 pA; (c) −1.5 V, 12 pA; (d) −1.5 V, 12 pA; (e) +1.5 V, 25 pA; (f) +0.45 V, 19 pA.

With respect to the underlying Pd(100) surface, the MoO_3_ layer forms a c(2 × 2) structure of planar MoO_2_ units (see DFT structure model in Figure 1, right panel), with each Mo atom terminated by an additional O atom.^36^ The latter ones are identified as the bright protrusions in the STM image. After depositing a small amount of 2H-Pc on the surface, additional features are visible in the STM image (Figure 2b), which are identified as flat lying 2H-Pc molecules. At this small coverage, the molecules stay isolated, however, they are predominantly adsorbed at defects, such as step edges and domain boundaries of the MoO_3_ film. If located within the domains, the molecules are easily displaced by the STM tip, as evident from their fuzzy appearance. This is the consequence of a rather weak molecule–substrate interaction, which is expected because of the oxygen termination of the film.

Figure 2c shows another region of the same surface after depositing a larger amount of 2H-Pc. Large areas of the MoO_3_ film are now covered by weakly bonded 2H-Pc, and it becomes practically impossible to properly image these regions at 77 K. However, at this coverage also large, 10 Å high molecular islands form, which display an ordered, stripe-like internal structure (Figure 2d). After mild annealing at 373 K to enhance the ordering, better resolved STM images reveal that the stripes consist of alternating rows of bright and dark protrusions (Figure 2e). Further deposition of 2H-Pc increases the fraction of such ordered islands. From this general growth behavior, we presume that the observed islands consist of 2H-Pc molecules in an upright, i.e. standing, adsorption geometry, which is consistent with previous observations of Pc monolayers on weakly interacting substrates.^51,52^

To support the conclusions from the STM results, we have additionally investigated the 2H-Pc film growth with NEXAFS at the N K-edge. Figure 2g shows the spectra for p- and s-polarized incident radiation for different 2H-Pc coverage. At the lowest coverage (0.07 ML) sharp π*-symmetry resonances are observed at 398.0 and 400.5 eV photon energy in p polarization; a characteristic π*-symmetry doublet is also observed in the 402–405 eV range, at the edge of the ionization threshold. These features are consistent with previous results and correspond to excitation into unoccupied molecular orbitals just above the Fermi level.^53^ In the corresponding spectrum taken with s polarization (Figure 2g), no π* features are observed, however, a broad σ*-symmetry feature is visible above 405 eV. This clearly shows that at the lowest coverage only flat-lying 2H-Pc molecules are present, in agreement with the STM results reported above. On increasing the 2H-Pc coverage (0.2 ML), the intensity of the π* resonances in the p polarization spectra is enhanced, but now a significant contribution of π*-symmetry resonances can be detected also in s polarization, indicating an increasing average tilt angle of the molecules away from the surface plane. For a complete monolayer of 2H-Pc, the intensity of the π* resonances in s-polarized mode by far exceeds that measured in p polarization, and the σ* resonances above 405 eV display an inverted dependence of NEXAFS intensity on the polarization geometry. From the intensity ratio of the π*-symmetry resonances measured in s and p polarization (so-called NEXAFS linear dichroism), we can determine the average molecular tilt off the surface according to the angular dependence rules for a 3-fold or higher substrate symmetry.^54^ The results of the analysis for the main peak at 398 eV are plotted in Figure 2h for all 2H-Pc coverages studied, showing that the average tilt angle increases with coverage until it stabilizes at 65–70° at monolayer coverage and above. Since the average tilt angle results from the combined contribution of a minority of flat-lying molecules anchored at defects and a majority of standing up molecules aggregated into island domains, we may confidently conclude that the standing up molecules are oriented closely normal to the surface.

Returning to the STM images of the ordered islands (Figure 2e), we conclude that standing 2H-Pc molecules are visible as two elongated lobes representing two isoindole arms of the molecule.^51^ The observed structure can then be explained by 2H-Pc forming stripes consisting of side-by-side stacked molecules where adjacent stripes exhibit a slip. We find that an oblique lattice with parameters *a* = 1.24 nm, *b* = 0.46 nm and γ = 100° fits the fundamental structural motif very well, where the molecular planes are rotated 20° relative to *a⃗*. Careful inspection reveals a second structural motif with an almost doubled *a* lattice vector (Figure 2f). It contains two molecules per unit cell and consists of 2H-Pc stripes arranged into a herringbone geometry between molecules in neighboring stripes. It is worth noting that the observed structural motifs are similar to the reported bulk crystal structures of phthalocyanines, such as the brickstone and the herringbone structure of the α phase.^55,56^ These results show that structural motifs typical for bulk phthalocyanine crystals are formed already in the first monolayer of 2H-Pc on the MoO_3_ thin film, indicating the strong influence of intermolecular interactions and a comparably weak bonding to the substrate. With the details of the growth of 2H-Pc on MoO_3_ obtained from STM and NEXAFS, we propose that the different slopes of the ΔΦ in the monolayer regime (Figure 1) can be explained by the transition from a coverage regime with predominantly flat-lying 2H-Pc molecules adsorbed at defects, to a regime where upright-standing 2H-Pc molecules aggregate into island domains.

While the weak molecule–substrate interaction favors the formation of islands of upright 2H-Pc molecules, flat lying molecules would be more convenient when aiming for the determination of the charge state with STM. This is because the population of orbitals and thus the charge state of the molecules would become evident from the orbital appearance in STM.^30^ While isolated flat molecules are present at low coverage (Figure 2b), their high mobility on terraces prevents the discrimination of intramolecular features in STM imaging at 77K, whereas flat molecules immobilized at defects display a distorted electronic structure. To stabilize flat molecules on regular, defect-free MoO_3_ patches, a different preparation strategy, which eliminates the influence of the line defects (domain boundaries, Figure 2a) must therefore be pursued. Since the line defects are the most favorable binding sites, we employed in this case a linear molecule, namely pentacene (5A), to decorate the domain boundaries prior to the adsorption of 2H-Pc. An example of the 5A-covered MoO_3_ surface is shown in Figure 3a. When, as in this case, the 5A coverage is properly adjusted, (almost) all line defects are saturated with 5A, and square patches of regular, defect-free MoO_3_ remain. We presume that the 5A molecules act as diffusion barriers for the subsequently adsorbed 2H-Pc and allow to trap flat-lying 2H-Pc in the confined MoO_3_ areas.

**Figure 3:**
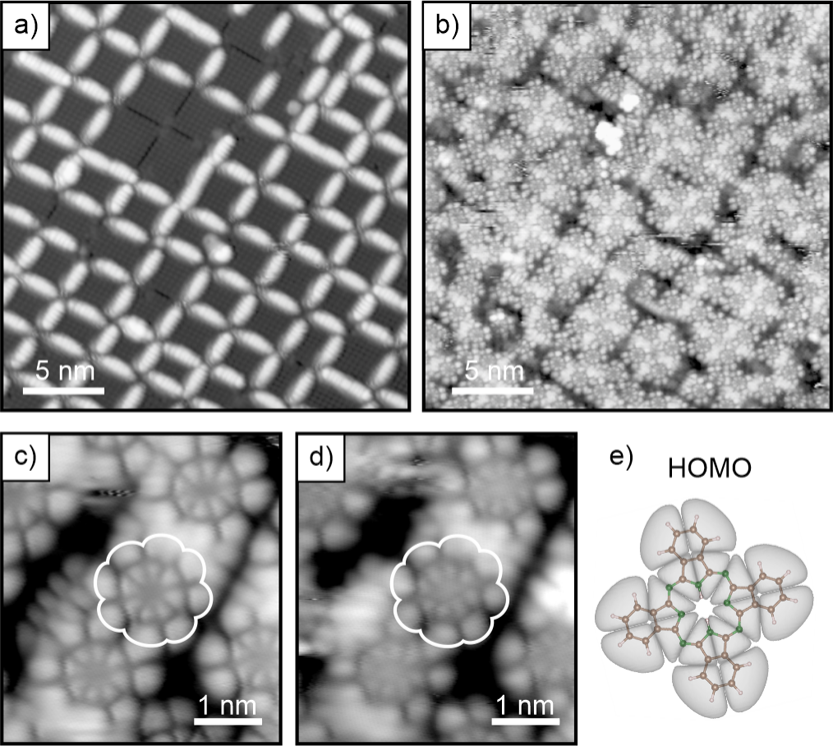
STM image of MoO_3_/Pd­(100) with (a) pentacene deposited to cover the antiphase domain boundaries, and (b) subsequently deposited 2H-Pc. (c, d) Detail STM images of flat 2H-Pc molecules located on defect-free MoO_3_ areas taken at (c) positive bias (empty states, *V*_b_ = +0.5 V) and (d) negative bias (filled states, V_b_ = −1.5 V). (e) Calculated HOMO of the free 2H-Pc molecule. Tunneling conditions: (a) +1.3 V, 14 pA; (b) +1.3 V, 8 pA; (c) +0.5 V, 10 pA; (d) −1.5 V, 10 pA.

Indeed, as shown in Figure 3b, this procedure turned out to be successful. On average four 2H-Pc molecules are stabilized within one MoO_3_ domain. Already from the large-scale image in Figure 2b, it is apparent that orbital fingerprints of the flat-lying 2H-Pc molecules can be resolved. A more detailed view of the 2H-Pc molecules is provided in Figure 3c,d, taken at +0.5 and −1.5 V bias voltage, respectively. Both images reveal a similar orbital structure and a comparison with 2H-Pc molecular orbitals suggests that the imaged electron density distribution emerges from the molecular HOMO (Figure 3e) in both cases. The appearance of the HOMO in the empty states image (+0.5 V) indicates a partial emptying of the HOMO due to electron transfer from the molecule into the substrate, which is in line with the expectations from the work function measurements (Figure 1). If the HOMO had been completely emptied, one would expect the HOMO–1 as the first state to appear in the filled states. The fact that we also see HOMO appearance here (Figure 3d) strongly suggests that we observe a SOMO-SUMO pair formed as a consequence of single integer charge transfer.

To further investigate the charge transfer characteristics of the 2H-Pc/MoO_3_ system, we utilize DFT employing a repeated slab approach. To this end, we construct structural models derived from the geometries experimentally observed for flat-lying and upright standing 2H-Pc structures (Figure 4a,b) and optimize their respective geometries. In our analysis of the DFT results, we particularly focus on the charge redistribution upon adsorption and the molecular electron density of states. In both cases, electron density is depleted from the molecules (blue lobes in Figure 4a,b), while electron accumulation occurs predominantly on the terminating oxygen atoms of the MoO_3_ film (yellow lobes in Figure 4a,b). The situation described is opposite to the previously investigated integer charge transfer processes for molecules on MgO(001)/Ag(001), where the electron was transferred from the MgO-Ag interface into the organic molecules.^27^

**Figure 4:**
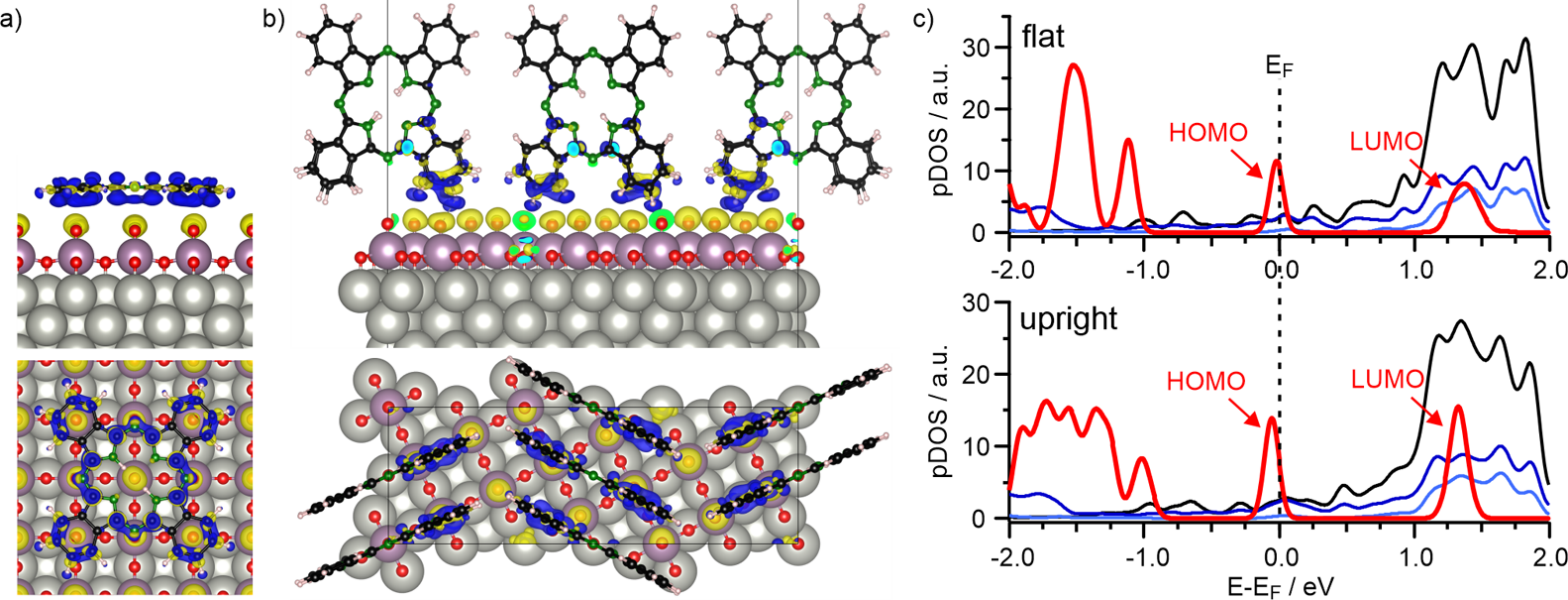
Results of DFT calculations: (a, b) Side and top views of 2H-Pc on MoO_3_/Pd­(100) in flat (a) and upright (b) adsorption geometry. Charge flow upon adsorption is represented by blue (charge depletion) and yellow (charge accumulation) lobes, respectively. (c) Partial density of states (pDOS) of the 2H-Pc/MoO_3_/Pd­(100) systems with flat (top) and upright (bottom) adsorption geometry. (pDOS contributions: C­(p_*z*_): red; Mo 3d: black; O_in‑plane_: dark blue; O_top_: light blue.).

For both flat and upright 2H-Pc on MoO_3_, the DFT results suggest that the charge redistribution is largely restricted to the molecule-oxide interface. Moreover, the top-view of the charge density difference map of the flat-lying molecule in Figure 4a clearly resembles the shape of the molecular HOMO (cf. Figure 3e), confirming that the charge is uniformly extracted from the HOMO. However, for the upright standing 2H-Pc (Figure 4b), the DFT result suggests that the charge depletion is nonuniform across the molecule and strongly localized to the interface with the oxide. Importantly, analysis of the change of the electrostatic potential across the interface reveals a theoretical work function change of −2.0 eV upon formation of an upright standing 2H-Pc layer on MoO_3_, which is due to the formation of a charge transfer dipole (Supporting Information SI.4). This is in perfect agreement with the experimentally determined work function change upon formation of a monolayer of upright molecules (Figure 1). Furthermore, DFT predicts a ΔΦ of −1.2 eV for the flat adsorption geometry (Supporting Information SI.4). This value also agrees well with the experimental ΔΦ at a coverage of ∼0.2 ML, which marks the transition from flat to upright adsorption geometries (Figure 1).

The charge transfer mechanism outlined above becomes also evident when analyzing the partial density of states (pDOS) shown in Figure 4c. For both, flat-lying and upright-standing geometries, the molecular HOMO spans the Fermi level, which is typical for Fermi level pinning as a result of charge transfer at the interface. It should be noted that, while our experimental results suggest the occurrence of integer charge transfer, which leads to splitting of the HOMO into a SOMO/SUMO pair with the corresponding peaks located below and above the Fermi level, respectively, our theoretical models do not show such a level splitting. This difference is related to technical limitations of our present DFT model. First, our calculations have been performed in a non spin-polarized approach. Second, the theoretical description of integer charge transfer and the appearance of a SOMO/SUMO would require a hybrid functional with a substantial fraction of exact exchange, rather than the presently used semilocal functional, and presumably also a larger supercell owing to the required symmetry breaking.^50^ Unfortunately, considering all these effects is still out of reach for the large cells studied here.

Experimentally, the DOS around the Fermi level can be probed using scanning tunneling spectroscopy, which we used to measure the DOS of both upright standing and flat-lying 2H-Pc (Figure 5). For the flat molecules (red curve), a large gap of Δ = 1.5 eV exists around the Fermi level and the first states appear at a bias voltage of −1.0 V in the occupied region and at +0.5 V in the unoccupied region. According to the STM images in Figure 3c,d, both states have HOMO characteristics and are therefore assigned to the corresponding SOMO and SUMO of the singly positively charged 2H-Pc. Note that a SOMO-SUMO gap of similar size has been observed for flat lying pentacene molecules on MgO(001)/Ag(001), which exhibits reversed charge transfer (from substrate to molecule).^27^ Turning to the upright 2H-Pc molecules, a significant gap reduction is noted (blue curve). The states appear at −0.15 and +0.25 V bias voltage, yielding a SOMO-SUMO gap of only 0.4 eV.

**Figure 5:**
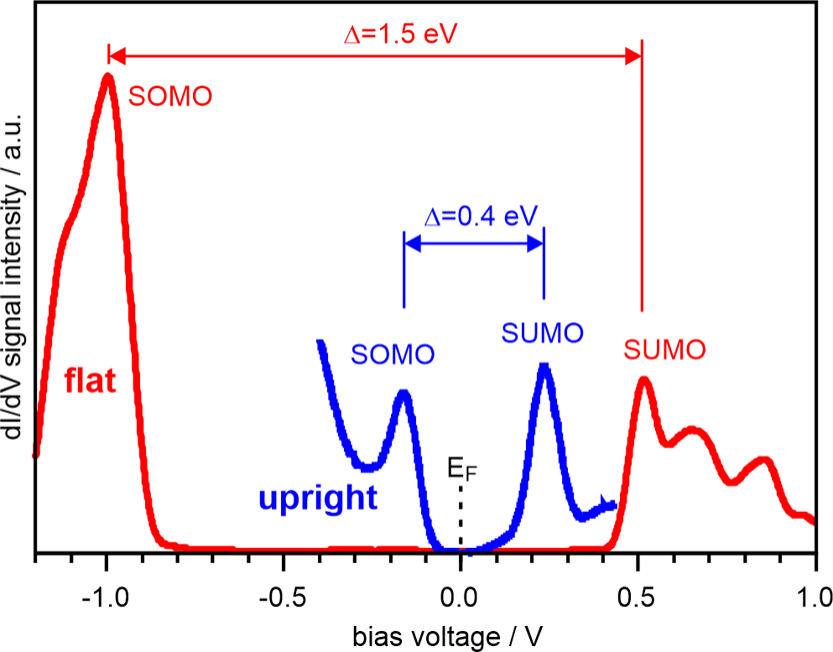
Experimental d*I*/d*V* spectra recorded atop 2H-Pc molecules with flat (red) and upright (blue) adsorption geometry on MoO_3_/Pd­(100). Tip stabilization: red: +1.5 V, 10 pA; blue: +0.5 V, 10 pA.

Orientation-dependent variations of the position of energy levels of organic semiconductors at interfaces have been observed previously. Compared to a free molecule, the ion state in molecules contained in molecular layers on surfaces is screened by charge carriers of the neighboring molecules and by the image charge in the metal. Both effects lead to a reduction of the ionization potential and an increase of the electron affinity, and thus to a reduction of the gap.^57^ Additionally, intrinsic dipoles in molecules affect the electrostatic potential. Particularly for planar π-conjugated molecules this leads to orientation-dependent changes of the ionization potential, with flat adsorption geometries typically exhibiting a larger ionization potential due to the presence of the negatively charged π electron cloud on the vacuum side.^58^ These effects are well-described for uncharged molecules at interfaces.

Here, we show a strong orientation-dependent size change of the SOMO/SUMO gap of singly positively charged 2H-Pc. Considering the work functions and SOMO binding energies for the two geometries, ionization potentials for flat and upright 2H-Pc are found to be 6.5 and 4.9 eV, respectively. The difference of 1.6 eV is significantly larger than that observed for uncharged, flat and upright molecules (e.g., 0.6 eV in ref 58). While for the uncharged case this effect has largely been ascribed to the intrinsic molecular dipole, in the present case, while these dipoles may contribute to the observations, the charge transfer and screening appear more important. First, the electron transfer induces interface dipoles that reduce the work function to a different extent for flat and upright molecules. Second, the SOMO/SUMO gap arises from the Coulomb energy required to add an electron to the SOMO and is formally derived from the difference between the ionization potential and the electron affinity of the molecular cation. A comparison of calculated SOMO/SUMO gaps for a single 2H-Pc^+^ and molecular clusters of up to eight 2H-Pc^+^ molecules stacked together shows a significant reduction of the gap from 3.07 eV (single molecule) to 1.32 eV (cluster of 8 molecules), which can be solely ascribed to screening (Supporting Information SI.5). While the calculated gaps are significantly larger than the observed ones due to not taking the presence of the surface into account, the results show that intermolecular screening can easily account for the strong SOMO/SUMO gap reduction when going from flat to upright adsorption geometry.

## 4 Conclusions

In this study, we have investigated the mechanism of charge transfer at oxide/organic interfaces using 2H-phthalocyanine (2H-Pc) adsorbed on ultrathin MoO_3_ films grown on Pd(100) as a model system for a technologically important interface. Combining work function measurements, STM/STS, NEXAFS, and DFT, we provide direct spectroscopic evidence for integer charge transfer from the HOMO of 2H-Pc into MoO_3_, leading to the formation of singly charged molecules and the emergence of distinct SOMO–SUMO states.

Our results demonstrate that the magnitude of the SOMO–SUMO gap is strongly dependent on the adsorption geometry and local screening environment. Upright-standing 2H-Pc molecules exhibit a narrow gap (∼0.4 eV), attributed to enhanced intermolecular screening and reduced molecule–substrate coupling. In contrast, flat-lying molecules, stabilized via pentacene passivation of MoO_3_ line defects, show a significantly larger gap (∼1.5 eV), reflecting diminished electronic screening and stronger confinement of charge.

These findings provide atomic-scale insights into the nature of ICT from molecules supported on high work function oxide substrates. Importantly, they underline the critical role of molecular geometry, interface morphology, and local screening in determining charge transfer energetics.

## Supplementary Material



## Notes

Details about sample preparation, work function measurements, 2H-Pc coverage determination, molecular tilt angle determination, charge density difference plots, and computation of the fundamental gap of 2H-Pc cation clusters (PDF)
