# Supplementary material for: Microscopic Origin of Charge Transfer at the Organic Semiconductor/MoO3 Hybrid Interface
Source: J Phys Chem C Nanomater Interfaces. 2025 Oct 7;129(41):18822–30. doi: 10.1021/acs.jpcc.5c05641 (PMC12536391; doi:10.1021/acs.jpcc.5c05641)
Supplement: Supplementary file 1 [file jp5c05641_si_001.pdf]

Supporting Information

Microscopic Origin of Charge Transfer at the Organic Semiconductor/MoO_3 Hybrid Interface

Max Niederreiter,[1] Maximilian Lasshofer,[1] Francesco Presel,[1] Giovanni Zamborlini,[1] Luca Floreano,[2] Luca Schio,[2] Nadia C. Mösch-Zanetti,[3] Svetlozar Surnev,[1] Peter Puschnig,[1] Martin Sterrer[1]

[1]University of Graz, NAWI Graz, Institute of Physics, Universitätsplatz 5, 8010 Graz, Austria.

[2]CNR-IOM, Laboratorio TASC, Basovizza SS-14, Km 163.5, Trieste 34012, Italy.

[3]University of Graz, NAWI Graz, Institute of Chemistry, Universitätsplatz 5, 8010 Graz, Austria.

SI.0. Sample preparations

The XPS and NEXAFS measurements presented where all performed at the ALOISA beamline at Elettra Sincrotrone Trieste, and are comprised of two preparation series, where first MoO_3 films were prepared and then 2H-Pc was deposited on top in steps. As a consequence, two MoO_3 films serve as reference for work function and coverage determinations, but we restrict ourselves to only show one in plots for the sake of clarity. These two oxide films have practically the same work function (WF) (ΔWF ~ 0.05 eV). The 2H-Pc films of coverage 0.2 ML and 0.8 ML were prepared on the first oxide film, while those of coverage 0.07 ML, 1 ML and 1.5 ML were prepared on the second oxide film.

SI.1. XPS work function measurements

The WF of all preparations, molecular films and oxide films, was determined using XPS by measuring the secondary electron cut-off (SEC). For this, a bias of 30 V was applied to the sample and a photon energy of 140 eV was used, the resulting XPS spectra for all molecular films and one of the two MoO_3 films are shown in Figure S1. Here, only one of the two MoO_3 spectra is shown for clarity since these two films have almost the same WF anyway (ΔWF~0.05 eV). In order to acquire an accurate value for the SEC, a linear fit was performed for all spectra in the region of steepest intensity decline, and the intersection of this fit function and the background intensity was used as the SEC. The relative shifts in WF are then the differences in SEC. To arrive at absolute values for the WF the value for Pd(100) was fixed to 5.6eV.[1]

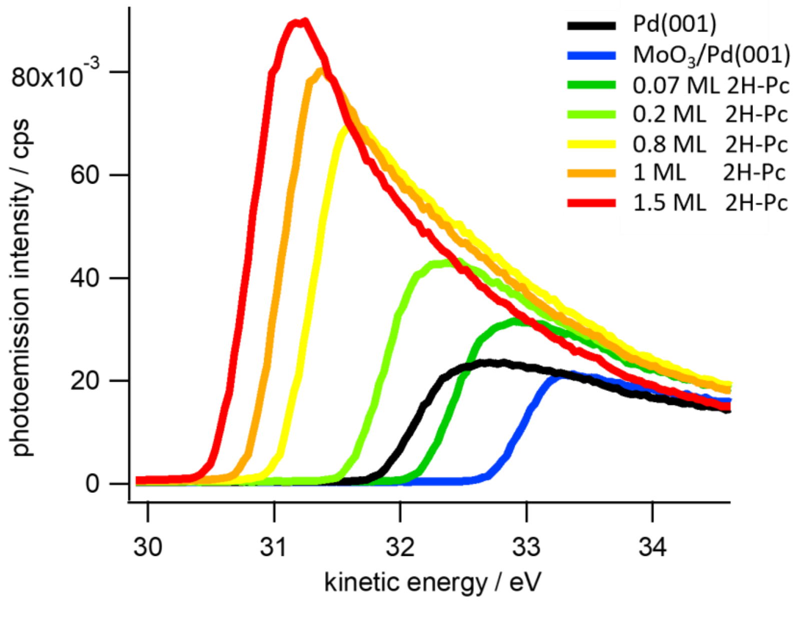

Figure S1. secondary electron cut-offs for all 2H-Pc film preparations and one of the two MoO_3 preparations, measured using XPS at hv = 140 eV (ΔE = 110 meV), U_{bias} = 30 V

SI.2. 2H-Pc coverage determination using XPS

The coverage of 2H-Pc was determined using the attenuation of the Pd 3d peak after 2H-Pc deposition (Figure S2). In order to quantify the peak intensity, the spectra were fitted with mixed Gaussian-Lorentzian profiles (G = 76%, L = 24%), using a Shirley type background. Generally, the peak intensity follows the inelastic mean free path equation:

$$I(d) = I_{d=0} e^{-\frac{d}{\lambda(E)}}$$

where d is the film thickness in Å, λ(E) the mean free path of electrons of kinetic energy E in the film's material (6.7 Å at 190 eV electron kinetic energy for MoO_3 [2]). A coverage of 1 ML upright standing molecules is referred to a deposited amount equivalent to 15 Å 2H-Pc, in accordance with 2H-Pc depositions performed in the STM chamber.

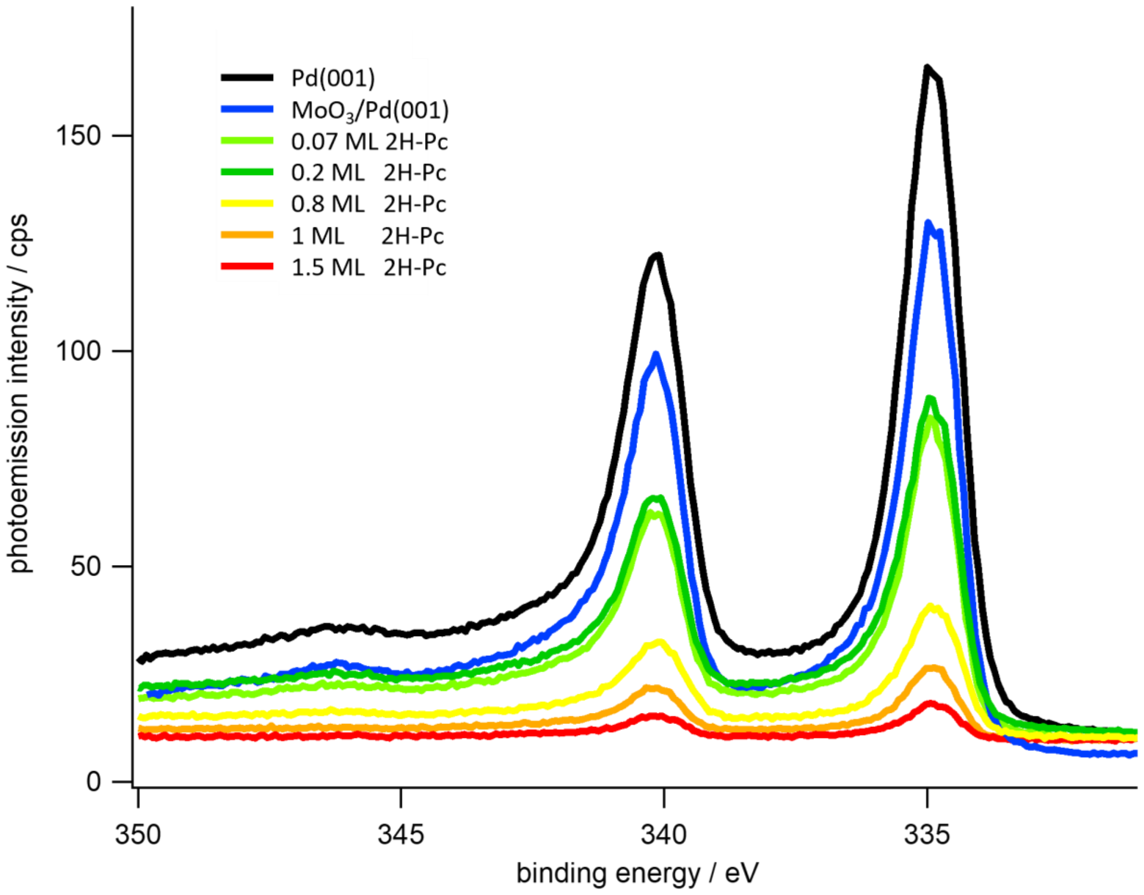

Figure S2. Pd 3d peaks measured using XPS at hν = 525 eV (ΔE = 160 meV). Note that the Pd(100) and MoO_3 spectra are only shown for the first preparation series. This is also the reason why the 0.2 ML 2H-Pc spectrum shows a higher intensity here than the 0.07 ML 2H-Pc spectrum, since they belong to different series with different baseline intensities after oxide preparation.

SI.3. Molecular tilt angle measurements using NEXAFS

The molecular tilt angle w.r.t. to the substrate surface was determined using the dichroism of π*-transitions in N k-edge spectra. Since the substrate exhibits four-fold rotational symmetry, the intensity of these transitions in TE- and TM-polarization can be described by the following equations[3]:

$$I_{TM} = \frac{1}{3}\left[1 + \frac{1}{2}(3cos^2\theta - 1)(3cos^2\alpha - 1)\right]$$

$$I_{TE} = \frac{1}{2}sin^2\alpha$$

Here, θ is the x-ray angle of incidence, which at ALOISA beamline is a constant 6°.[4] The angle α is defined as the angle between the surface normal and the axis of the π*-orbital into which excitations occur. In the present case, this also happens to be the molecular tilt angle, i.e. the angle between the molecular plane of 2H-Pc and the substrate surface. Given the fixed angle θ, the dichroism $\frac{I_{TE}}{I_{TM}}$ can be solved for α, these values are shown in Figure 2h in the main text. The general dependence of $\frac{I_{TE}}{I_{TM}}$ on the angle α as described by the above equations is shown in Figure S3.

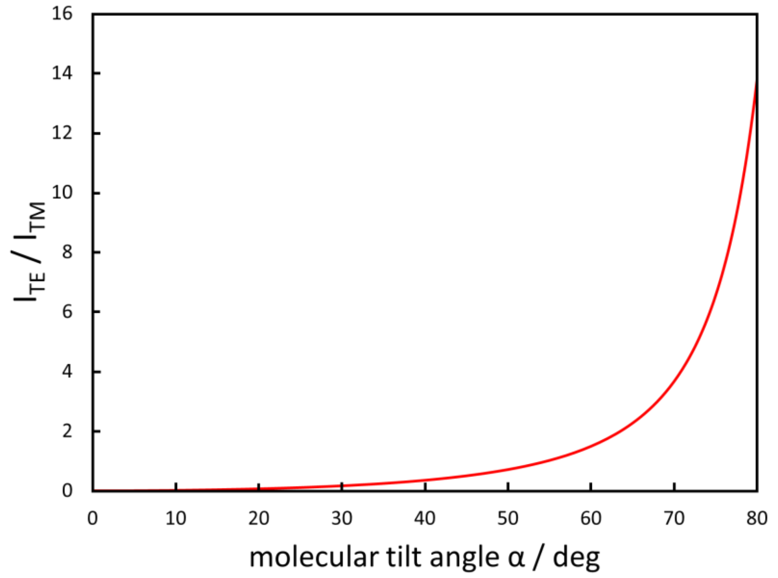

Figure S3. Polarization dependent dichroism $\frac{I_{TE}}{I_{TM}}$ for the given experimental setup, shown as a function of tilt angle α.

The formalism used here applies to the case of full linear polarization of the incident x-rays (P = 1), at the ALOISA beamline the actual polarization is P = 0.98. However, the practical significance of this discrepancy is negligible and this effect is therefore not taken into account in this analysis. For low 2H-Pc coverages we find very low molecular tilt angles using NEXAFS, e.g., α = 16.1° at 0.07 ML. It should be noted that at such low angles the uncertainty is relatively large, since the intensity of I_{TE} is quite low and therefore more easily affected by noise. The consequence of this can be seen easily in Fig. S3, where small variations of $\frac{I_{TE}}{I_{TM}}$ can lead to large variations of the tilt angle when $\frac{I_{TE}}{I_{TM}}$ itself is small. Ultimately, this means very small molecular tilt angles determined with this method should not be interpreted as numerically accurate estimates of the physical tilt angle, but should rather be seen as a qualitative indicator that the molecules lie flat on the surface.

SI.4. Charge density difference plots

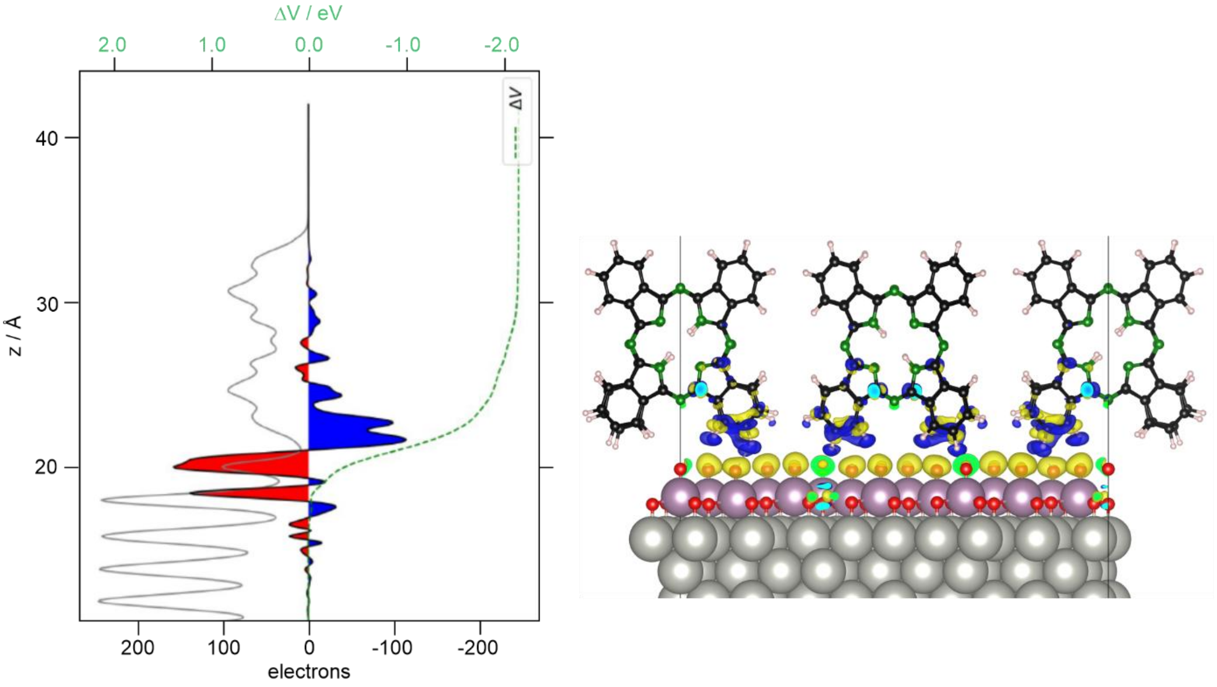

Figure S4.1. Charge density difference and resulting potential difference induced by adsorption of upright standing 2H-Pc on MoO_3/Pd(100).

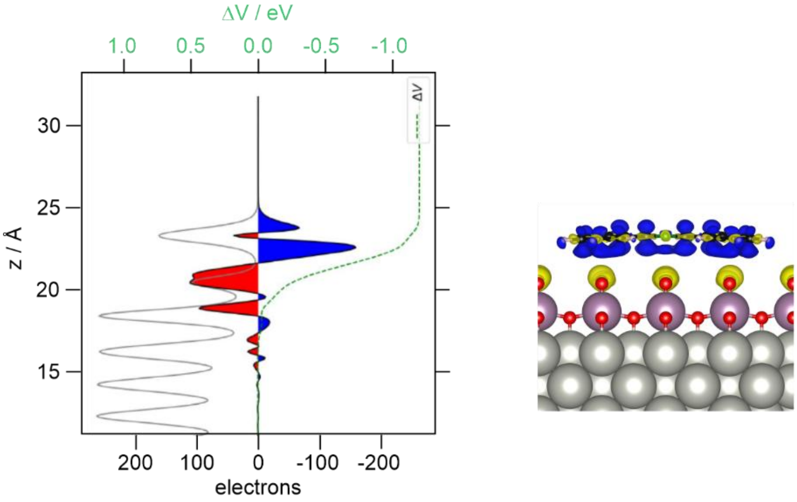

Figure S4.2. Charge density difference and resulting potential difference induced by adsorption of flat-lying 2H-Pc on MoO_3/Pd(100).

SI.5. Reduction of the fundamental gap of the 2H-Pc cation induced by intermolecular screening.

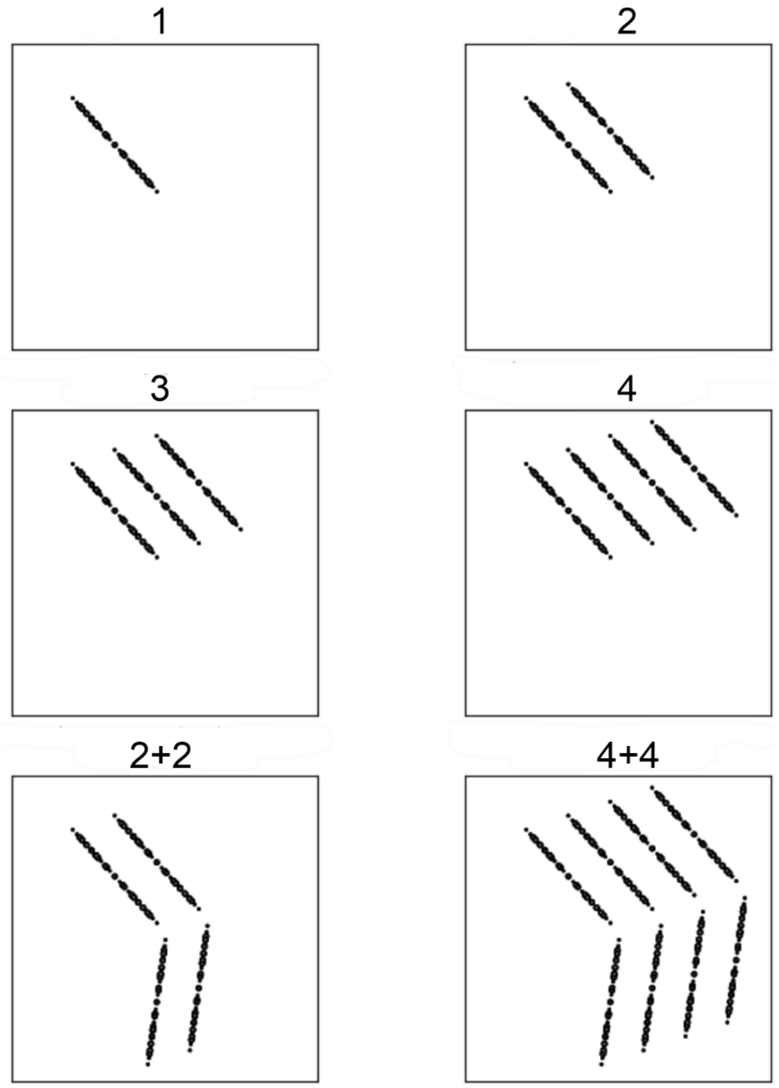

Figure S5. Models of free-standing 2H-Pc clusters used to calculate the ionization potential (IP) and electron affinity (EA) of the 2H-Pc$^+$ cation.

Table S5. Ionization potential (IP), electron affinity (EA) and fundamental gap (Eg) of the cationic state of 2H-Pc clusters.

Cluster	IP(N-1)	EA(N-1)	E_g(N-1)
1	9.48	6.41	3.07
2	8.45	6.01	2.44
3	7.87	5.79	2.08
4	7.48	5.64	1.85
2+2	7.43	5.70	1.73
4+4	6.81	5.48	1.32

References

[1] Riviere, J. C., in: Work Function: Measurements and Results, in Solid State Surface Science. Vol. 1, Green, M., Ed., Decker, New York, 1969.

[2] QUASES-IMFP-TPP2M Ver. 3.0, Inelastic electron mean free paths calculated from the TPP-2M formula.

[3] Stöhr, J., NEXAFS spectroscopy. Springer Series in Surface Sciences, Springer Verlag: Berlin, Heidelberg, 1992; Vol. 25.

[4] Bavdek, G. et al. Pentacene nanorails on Au (110). *Langmuir* **2008**, *24*, 767-772.
